# Ultrasound-Guided Manual Therapy for the Infrapatellar Branch of the Saphenous Nerve Entrapment Presenting as Anterior Knee Pain: A Case Report

**DOI:** 10.7759/cureus.93051

**Published:** 2025-09-23

**Authors:** Toru Miyata, Masashi Kawabata, Daiki Watanabe, Kazuma Miyatake

**Affiliations:** 1 Rehabilitation Center, Sagamihara Kyodo Hospital, Sagamihara, JPN; 2 Department of Rehabilitation, Kitasato University School of Allied Health Sciences, Sagamihara, JPN; 3 Department of Orthopedic Surgery, Yokohama Minami Kyosai Hospital, Yokohama, JPN; 4 Department of Orthopedic Surgery, Yokohama City University, Yokohama, JPN

**Keywords:** anterior knee pain, case report, entrapment neuropathy, high-resolution ultrasound, infrapatellar branch of the saphenous nerve, ultrasound-guided manual therapy

## Abstract

The infrapatellar branch of the saphenous nerve (IPBSN) is the sensory branch that supplies the anteromedial knee. While iatrogenic injury following knee surgery is well documented, spontaneous or activity-related IPBSN entrapment is under-recognized and can mimic common extensor mechanism disorders.

Herein, we present the case of a male physical education teacher in his 20s who developed reproducible anterior knee pain with lunge movements after repeated basketball jumps. The initial clinical suspicion was patellar tendinopathy; however, high-resolution ultrasound (HRUS) revealed IPBSN entrapment at the sartorius penetration site. Manual decompression of the sartorius muscle immediately alleviated the pain. Subsequently, ultrasound-guided soft tissue mobilization was performed, resulting in complete resolution of pain.

This case highlights the diagnostic value of HRUS and introduces ultrasound-guided manual therapy as a potentially safe and effective conservative option for treating IPBSN presenting with anterior knee pain.

## Introduction

Anterior knee pain is a common complaint in orthopedic clinical practice. The well-known etiologies include extensor mechanism disorders, patellofemoral pain syndrome, osteoarthritis, medial meniscal injury, pes anserinus tendinopathy, and medial collateral ligament injury. Patellar tendinopathy is one of the most common anterior knee disorders, particularly in athletes involved in jumping sports [1]. It typically manifests as pain at the inferior pole of the patella during loading, with tenderness at the tendon insertion and characteristic ultrasound findings, such as tendon thickening and neovascularization.

In contrast to these disorders, entrapment neuropathy of the infrapatellar branch of the saphenous nerve (IPBSN) is an under-recognized cause of anterior knee pain. In nonsurgical populations, its true prevalence has not been established, with existing evidence limited to case reports and small case series [2-4]. In contrast, IPBSN injury has been reported at a high frequency (22.2-85%) in postoperative cohorts, particularly after arthroscopic knee surgery and anterior cruciate ligament (ACL) reconstruction [5-7]. Although IPBSN neuropathy is recognized postoperatively, its occurrence in nonsurgical cases remains poorly understood.

Anatomically, the IPBSN is a sensory branch of the saphenous nerve that supplies the skin and intra-articular structures of the anteromedial knee. Its course showed several normal anatomical variations in relation to the sartorius muscle, including the anterior, posterior, and penetration types. Notably, the “penetration type,” in which the IPBSN traverses the sartorius muscle, is reported in 21.6-70% of cases and may predispose to entrapment within the muscle [8-10]. In such cases, contraction of the sartorius muscle may lead to traction or compression of the nerve, potentially provoking pain [3].

Diagnosing IPBSN neuropathy is challenging because of the small caliber of the nerve and considerable anatomical variability. Localized tenderness and a positive Tinel’s sign, elicited by gentle tapping over the suspected nerve course to reproduce paresthesia in its distribution, may support clinical suspicion; however, these findings are nonspecific and influenced by anatomical variations [10]. Their diagnostic accuracy has not been systematically validated for IPBSN, and studies on other entrapment neuropathies, such as carpal tunnel syndrome, have demonstrated that Tinel’s sign has limited sensitivity when used alone [11]. From an imaging perspective, high-resolution ultrasound (HRUS) is regarded as the most valuable modality, allowing direct visualization of the IPBSN, identification of neuroma or entrapment, and alignment with clinical symptoms for diagnostic confirmation [10,12].

Herein, we report a rare nonsurgical case of anterior knee pain initially suspected to be patellar tendinopathy but with inconsistent clinical findings. HRUS enabled the diagnosis of IPBSN entrapment, and ultrasound-guided manual therapy resulted in marked symptomatic improvement. Given that reports on the conservative management of IPBSN neuropathy remain scarce, this case report aimed to demonstrate the diagnostic utility of HRUS and the therapeutic potential of ultrasound-guided manual therapy as a noninvasive treatment option.

## Case presentation

**Figure 1:**
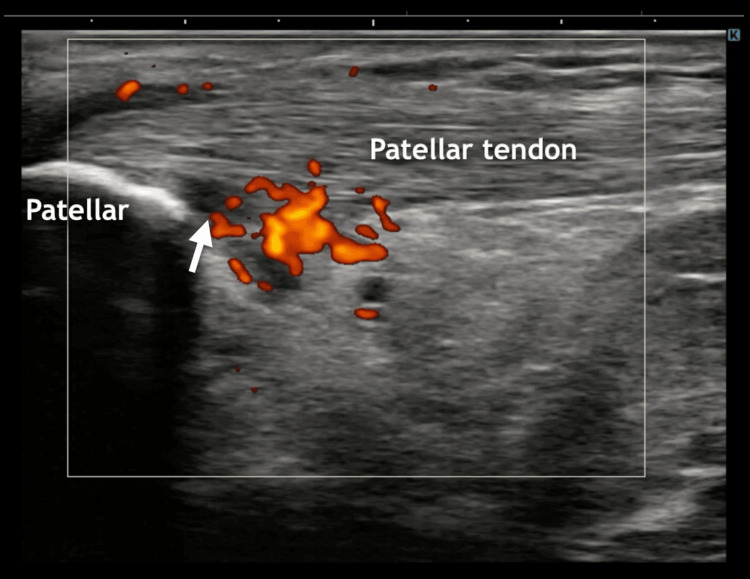
Long-axis ultrasound image of the left patellar tendon A slightly hypoechoic area with increased vascularity (arrow) is observed in the deep layer of the proximal portion of the patellar tendon. Despite these findings, no tenderness was observed at the site. The main body of the tendon maintained a normal fibrillar echotexture, suggesting preserved structural integrity.

**Figure 2:**
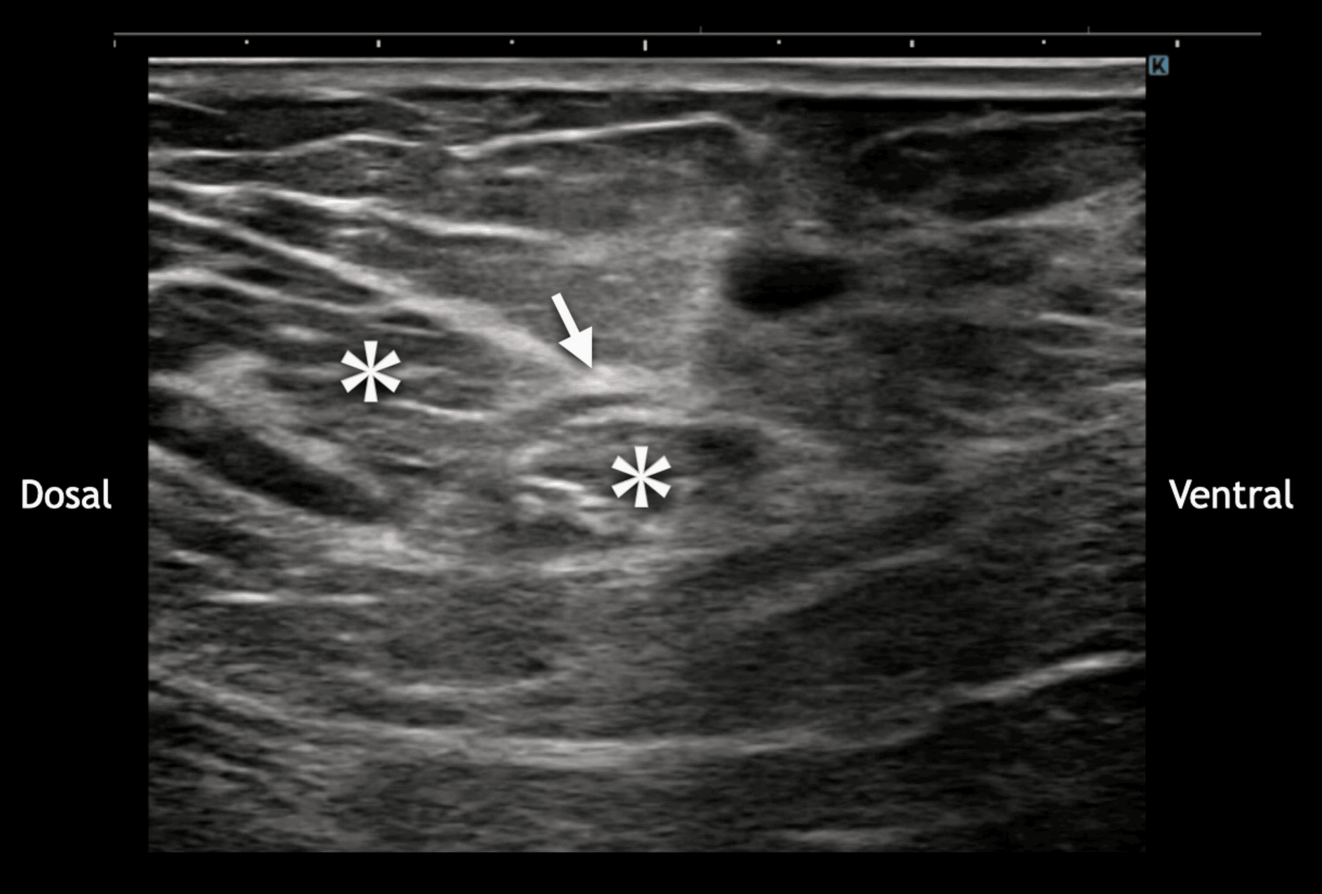
Short-axis ultrasound image of the sartorius muscle The infrapatellar branch of the saphenous nerve (IPBSN, arrow) penetrates the sartorius muscle (*). Localized tenderness was observed at this site, suggesting a potential entrapment point.

The patient was a male physical education teacher in his early 20s with a history of playing baseball during his school years. The knee joint range of motion was within normal limits. He had played basketball at a recreational level and had no significant medical history. The patient developed anterior pain in the left knee after repetitive jumping movements and subsequently presented to the orthopedic clinic. During the initial evaluation, the patient reported activity-related pain localized in the patellar tendon. Although other conditions, such as pes anserinus tendinopathy or synovial plica syndrome, could not be completely excluded, a working diagnosis of patellar tendinopathy was made based on the symptoms. Ultrasonography demonstrated a localized power Doppler signal in the deep layer of the patellar tendon (grade 1) [13]; however, the fibrillar architecture was preserved, and no structural abnormalities were identified (Figure 1). Notably, no tenderness was detected at the inferior pole of the patella, a typical feature of patellar tendinopathy. The maximal pain was slightly distal to the vascular signal on ultrasound, indicating a discrepancy between clinical symptoms and tendon imaging findings. Pain was assessed using the numerical rating scale (NRS). During lunging, the pain was reproducible (NRS: 7/10). The patient described it as pain “running across the patellar tendon.” As this complaint corresponded to the anatomical course of the IPBSN, IPBSN involvement was suspected. HRUS confirmed a neurovascular bundle penetrating the sartorius muscle at the proximal level with focal tenderness at this site (Video 1 and Figure 2), suggesting entrapment of the IPBSN. It should be noted that a confirmatory diagnostic nerve block was not performed.

**Video 1:** Identification of the IPBSN penetrating the sartorius muscle on ultrasound This video shows a transverse ultrasound scan of the sartorius muscle performed in the distal direction. As the probe advanced, a hyperechoic linear structure penetrating the muscle was observed. This structure corresponds to the infrapatellar branch of the saphenous nerve (IPBSN), confirming its anatomical relationship with the sartorius. This scanning technique helps identify potential entrapment points in patients presenting with anteromedial knee pain.

Dynamic assessment during lunging reproduced consistent pain (NRS, 7/10) radiating across the patellar tendon mediolaterally. The HRUS demonstrated that the IPBSN ran precisely across the pain locus. Proximal tracking identified a neurovascular bundle penetrating the sartorius muscle with focal tenderness at this site (Video 1 and Figure 2), suggesting entrapment of the IPBSN.

Intervention

**Figure 3:**
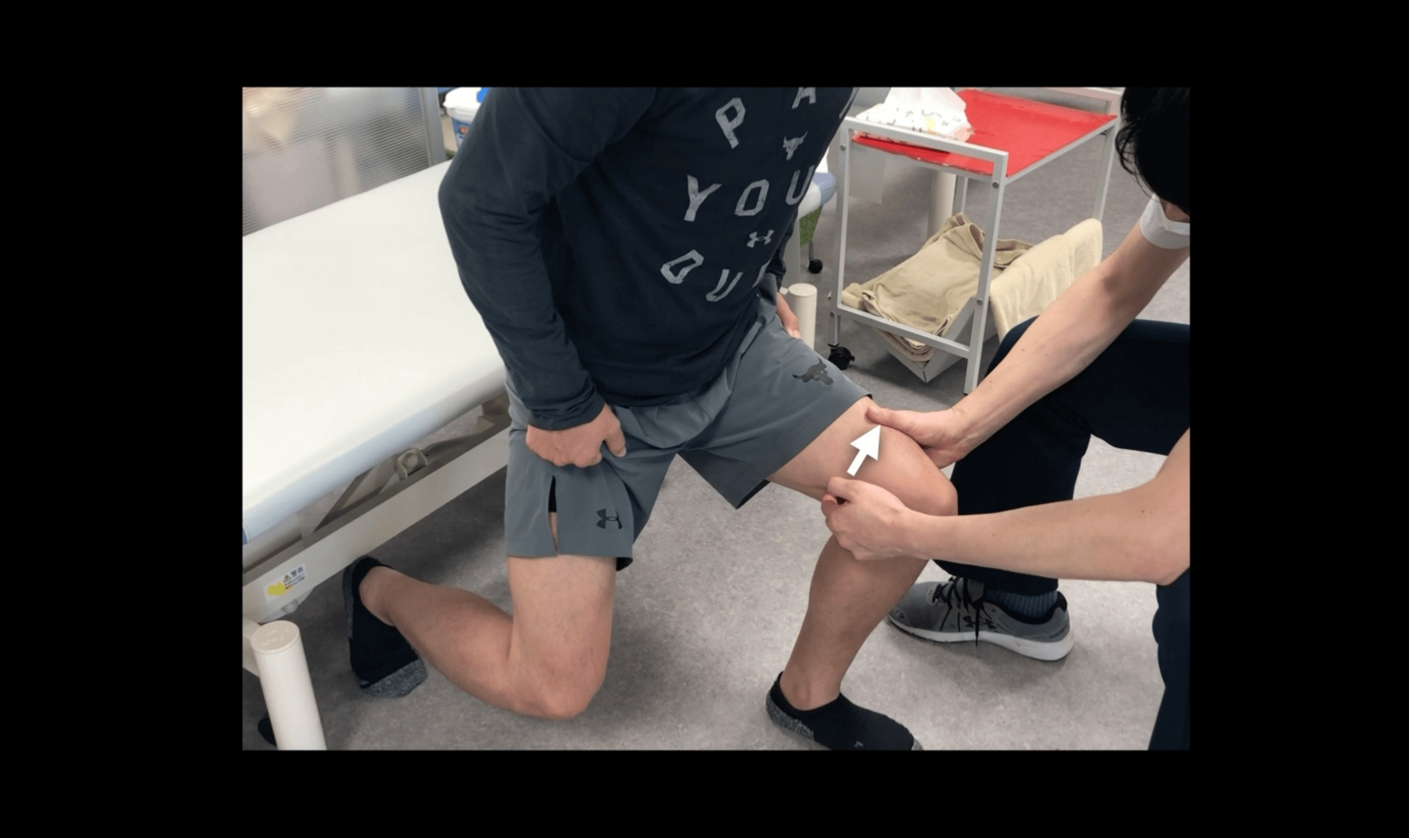
Sartorius Lift Test: manual decompression of the sartorius muscle The sartorius muscle is manually elevated from dorsal to ventral (arrow). Temporary alleviation of anteromedial knee pain during this maneuver served as a differential diagnostic sign of neuropathic pain related to the infrapatellar branch of the saphenous nerve (IPBSN). This test also functioned as a dynamic pain modulation assessment supporting the clinical suspicion of nerve entrapment.

**Figure 4:**
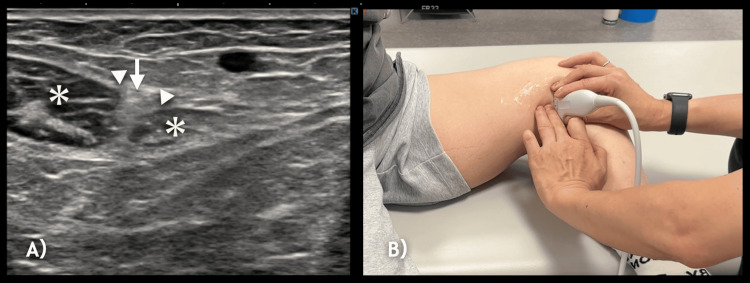
Ultrasound-guided mobilization of the sartorius muscle (A) Short-axis ultrasound image of the sartorius muscle (*). The infrapatellar branch of the saphenous nerve (IPBSN, arrow) can be seen penetrating the muscle. Gentle mobilization was applied in the direction indicated by the arrowhead (▲), along the posterior border of the sartorius. Care was taken to avoid the direct compression of the IPBSN during the procedure. (B) Photograph from another case showing the therapist’s hand positioning (image horizontally flipped for illustrative purposes).

Manual decompression of the sartorius muscle immediately alleviated pain during lunge testing (Figure 3 and Video 2), and this novel clinical maneuver was designated as the “Sartorius Lift Test.” Based on this finding, ultrasound-guided mobilization of the sartorius muscle at the point of IPBSN penetration was performed (Figure 4 and Video 3). This single intervention lasted for approximately five minutes under real-time ultrasound monitoring of tissue displacement, pressure vector, and depth. Care was taken to avoid direct compression of the penetrating IPBSN. Instead, the sartorius muscle was gently mobilized to promote nerve gliding without exacerbating the symptoms. The intervention was performed by a physiotherapist with three years of experience in musculoskeletal ultrasound at that time, and no adverse events were observed.

**Video 2:** Sartorius Lift Test for assessing entrapment of the IPBSN This video demonstrates the Sartorius Lift Test, a manual decompression technique used to assess entrapment of the infrapatellar branch of the saphenous nerve (IPBSN). In this maneuver, the sartorius muscle was gently lifted from the dorsal to the ventral direction while the patient lunged. Temporary relief of anteromedial knee pain during the maneuver supports the diagnosis of IPBSN-related neuropathic pain, which serves as a dynamic pain modulation tool and aids in differential diagnosis when imaging findings and clinical symptoms are incongruent.

**Video 3:** Ultrasound-guided manual mobilization of the sartorius: IPBSN interface This video demonstrates an ultrasound-guided manual mobilization targeting the soft tissue space between the sartorius muscle and the infrapatellar branch of the saphenous nerve (IPBSN). Using a short-axis view, gentle pressure is applied to the sartorius muscle while avoiding direct nerve compression, thereby promoting nerve gliding and reducing mechanical irritation around the IPBSN.

Outcome

Following this single intervention, the pain during lunging completely resolved, the NRS score decreased from 7 to 0 out of 10, and the single-leg squats were pain-free. Follow-up was conducted exclusively by telephone, without direct physical re-examination. At follow-up, the patient reported satisfaction with the treatment outcome, noting that residual symptoms were minimal and could be managed with self-care. There were no restrictions on his activities as a physical education teacher, and recreational basketball was possible with only mild discomfort. Functional recovery appeared favorable, but long-term outcomes could not be definitively confirmed.

## Discussion

A presentation of anterior knee pain caused by IPBSN entrapment, which is uncommon mainly because nonsurgical cases have been infrequently reported, was successfully treated using ultrasound-guided manual therapy. While IPBSN neuropathy is most often reported as an iatrogenic complication of knee arthroscopy or ACL reconstruction, nonsurgical cases may be misdiagnosed as hamstring contusions, fat pad irritation, or pes anserine bursitis [2,4]. Despite ultrasound evidence of minor patellar tendon changes, the clinical findings were atypical, underscoring the need to consider IPBSN neuropathy in the differential diagnosis of anterior knee pain, even in nonsurgical settings.

HRUS confirmed the concordance between the patient’s pain site and the IPBSN trajectory and demonstrated entrapment at the sartorius penetration site. Riegler et al. and Le Corroller et al. showed that HRUS reliably visualized the IPBSN from its origin to its distal branches in both cadaveric and volunteer studies [10,14]. Our findings are consistent with those of these previous reports. In addition, recent studies have emphasized that HRUS enables targeted procedures, such as perineural injections and hydrodissection, even for small-caliber nerves, underscoring its role not only as a diagnostic modality but also as a therapeutic tool [12].

Manual mobilization was applied precisely at the entrapment site identified by HRUS, resulting in the complete resolution of pain during functional movement. The defining feature of this case was that the ultrasound visualization of the IPBSN course and entrapment site directly guided the accurate application of manual therapy. Conventional management strategies for IPBSN include diagnostic nerve blocks and surgical decompression, both of which have been reported to provide significant pain relief [12]. However, these approaches are invasive and pose procedural risks. In contrast, ultrasound-guided manual therapy is noninvasive, reproducible, and safer than other invasive approaches. 

From a mechanistic perspective, neurodynamic interventions have been proposed to reduce neural mechanosensitivity and restore mobility [15]. In this case, gentle mobilization of the sartorius muscle may have reduced mechanical stress on the entrapped IPBSN; however, nerve gliding was not directly observed and should be regarded only as a possible explanatory mechanism. Ultrasound guidance further enhances the treatment accuracy and safety by allowing selective mobilization of the surrounding soft tissue without direct nerve compression. Although unrelated to peripheral nerve sliding, ultrasound-guided manual therapy has also been reported to improve tendon gliding after Achilles tendon repair [16], suggesting the broader applicability of ultrasound-assisted manual interventions. Therefore, ultrasound-guided manual therapy should be recognized as a promising conservative treatment option for entrapment neuropathies, including IPBSN.

IPBSN entrapment is often overlooked as a cause of nonsurgical anterior knee pain. Combining HRUS visualization with dynamic maneuvers such as the Sartorius Lift Test may improve diagnostic accuracy and broaden treatment options. Ultrasound-guided manual therapy is a practical, low-risk, conservative intervention. This approach integrates diagnostic imaging with targeted manual intervention and represents a potential paradigm shift in the conservative management of entrapment neuropathies.

Limitations

This was a single-case report, which limits the reproducibility and generalizability of the findings. Moreover, the causal link between improved nerve gliding and pain reduction could not be objectively quantified. The follow-up period was short, functional outcomes were assessed subjectively, and standardized parameters for ultrasound-guided mobilization were lacking, which may have hindered reproducibility. Further prospective studies are required to validate the clinical utility of ultrasound-guided manual therapy for peripheral nerve entrapment. In addition, potential placebo effects and lack of blinding cannot be excluded, which are inherent limitations of case reports.

## Conclusions

This rare case illustrates that anterior knee pain misattributed to patellar tendinopathy may, in some instances, be due to IPBSN entrapment. HRUS visualization and the Sartorius Lift Test contributed to an accurate diagnosis, whereas ultrasound-guided manual therapy provided effective noninvasive symptom relief. This report introduces a potential new diagnostic perspective and therapeutic strategy for anterior knee pain and offers important implications for future clinical practice. Further research is needed to clarify the true incidence of nonoperative IPBSN entrapment and evaluate the effectiveness of this management strategy.
